# Self-Similar Random Process and Chaotic Behavior In Serrated Flow of High Entropy Alloys

**DOI:** 10.1038/srep29798

**Published:** 2016-07-20

**Authors:** Shuying Chen, Liping Yu, Jingli Ren, Xie Xie, Xueping Li, Ying Xu, Guangfeng Zhao, Peizhen Li, Fuqian Yang, Yang Ren, Peter K. Liaw

**Affiliations:** 1 Department of Materials Science and Engineering, The University of Tennessee, USA; 2 School of Mathematics and Statistics, Zhengzhou University, China; 3 Department of Chemical and Materials Engineering, University of Kentucky, USA; 4 X-ray Science Division, Argonne National Laboratory, Argonne, Illinois, 60439, USA

## Abstract

The statistical and dynamic analyses of the serrated-flow behavior in the nanoindentation of a high-entropy alloy, Al_0.5_CoCrCuFeNi, at various holding times and temperatures, are performed to reveal the hidden order associated with the seemingly-irregular intermittent flow. Two distinct types of dynamics are identified in the high-entropy alloy, which are based on the chaotic time-series, approximate entropy, fractal dimension, and Hurst exponent. The dynamic plastic behavior at both room temperature and 200 °C exhibits a positive Lyapunov exponent, suggesting that the underlying dynamics is chaotic. The fractal dimension of the indentation depth increases with the increase of temperature, and there is an inflection at the holding time of 10 s at the same temperature. A large fractal dimension suggests the concurrent nucleation of a large number of slip bands. In particular, for the indentation with the holding time of 10 s at room temperature, the slip process evolves as a self-similar random process with a weak negative correlation similar to a random walk.

High-entropy alloys (HEAs)^1^,^2^,^3^,^4^,^5^,^6^,^7^,^8^,^9^,^10^,^11^,^12^,^13^,^14^,^15^ found in 1990’s have attracted great attention due to their unique structural properties. HEAs are defined as the multi-principal component alloys, including five or more principal elements, with molar ratios ranging from 5~35 atomic percent (at. %). HEAs are different from conventional alloys with one or two major components, i.e., the addition of small amounts of other alloying elements into alloys in order to enhance physical properties, such as Al-based alloys and Co-Ni-based superalloys. Recently, it was reported that ternary and quaternary alloys can also be regarded as HEAs, such as ZrNbHf^16^ and WNbMoTa^17^. Specifically, with a large number of elements, HEAs soon form solid solutions with structures, including body-centered-cubic (BCC) structure^3^,^17^,^18^,^19^, face-centered-cubic (FCC) structure^3^,^20^,^21^,^22^, or hexagonal-closed-packed (HCP) structure^3^,^23^,^24^,^25^, rather than complicated intermetallic compounds, which could be attributed to the large configurational entropy. In addition, HEAs possess superior properties, such as the enhanced yield strength^26^, good resistance to wear and fatigue^15^,^22^,^27^ and corrosion^28^,^29^, remarkable fracture-toughness at cryogenic temperature^4^, and excellent properties at elevated temperatures^30^.

Nanoindentation has been used to probe the mechanical properties of materials on micron and submicron scales, such as contact modulus, hardness, creep parameters^31^,^32^,^33^,^34^, and residual stresses^35^. Although nanoindentation has become a great technique for the measurement of mechanical properties on small scales, it may have even greater importance as a method to experimentally study the fundamental knowledge controlling the physics of materials, such as staircase-like shapes observed in the displacement-time curves of bulk metallic glasses at low deformation rates^36^.

Serrated flows in the plastic regime of HEAs have been widely observed in compression and nanoindentation tests. In the compressive tests of HEAs, it was shown that the physical origin of this intermittent flow was with a function of testing temperature, composition, phase formation, strain rate, and so on^3^,^12^,^37^,^38^,^39^,^40^. For example, Zhang *et al*. found that the serration is greater at low temperatures with a strain rate of in 10^−3^ *s*^−1^ than 10^−1^ *s*^−1 ^ Zhang *et al*.^3^; analyzing the sizes of serration, Dahmen *et al*.^12^ found that increasing temperature led to the transition of the serrated stress-strain curves from type-A to B to C PLC-band^41^ (characterized by almost periodically-recurring large slips). Nevertheless, how the serrations change with external conditions, and the physical mechanism in nanoindentation of high-entropy alloys are still unclear. In order to answer the above questions, we use statistical and dynamic analyses to extract the hidden information of the intermittent flow in the nanoindentation of a high-entropy alloy, Al_0.5_CoCrCuFeNi, at various holding times and temperatures.

## Experimental Details

The Al_0.5_CoCrCuFeNi (in atomic percentage) alloy was prepared by arc-melting a mixture of principal elements of high purity [>99.9 weight percent (wt.%)] in a Ti-gettered high-purity argon atmosphere. The processes of melting and solidification were repeated at least five times to achieve high homogeneity. The molten alloy was, then, drop-cast into a water-cooled copper mold of 2 mm in diameter. A disk of 2 mm in thickness, which was cut from the as-cast Al_0.5_CoCrCuFeNi HEA rod, was mechanically ground and polished to obtain two parallel surfaces of a mirror quality to eliminate surface effects.

High-energy synchrotron X-ray diffraction (XRD) was performed at the advanced photon source (APS), using the 11-ID-C beam-line located at the Argonne national laboratory to obtain the initial diffraction patterns of the sample for structural characterization.

Nanoindentation tests were carried out in a Nano Test Vantage (Micro Materials). A diamond Berkevich indenter with a nominal tip radius of ~50 nm was used. The machine compliance was calibrated to be 0.30 nm/mN. The nanoindentation test was performed under the mode of load control with the peak load of 100 mN. Both the loading rate and unloading rate were 10.00 mN/s. Room temperature and 200 °C were chosen to study the effect of temperature on the indentation deformation. For each temperature, three different holding times of 5, 10, and 20 s at the peak load were used. At least 3 indents were performed for each indentation condition. The penetration depth and indentation load were used in the characterization of the near-surface mechanical behavior of the Al_0.5_CoCrCuFeNi HEA.

There is a heat shield to ensure no heat flowing into the loading head with sensitive components and a separate active heat controller for both the cement -sample stage and indenter to limit the heat flow during indentation. There is also a water-cooled heat shield to contain the radiant heat from the hot stage. The procedure of automatic multiple stages was used to heat the sample and indenter to the pre-determined temperature with the highest stability. Both the sample and indenter were heated at 1.6 °C per minute to avoid any thermal shock. During the indentation, a corrected thermal drift rate was calculated, using the post indentation data. For each indentation, the thermal drift rates were measured three times. All the displacements for the same test condition were corrected, using the obtained thermal drift according to the time when they were recorded. For all the nanoindentation at the temperature of 200 °C, the calculated thermal drift rates were less than 0.9312 nm/s.

**Figure 1:**
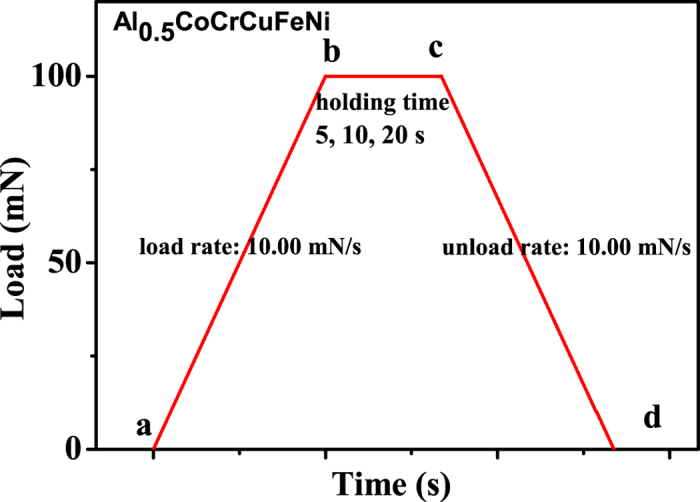
Schematic of indentation load vs. time.

**Figure 2:**
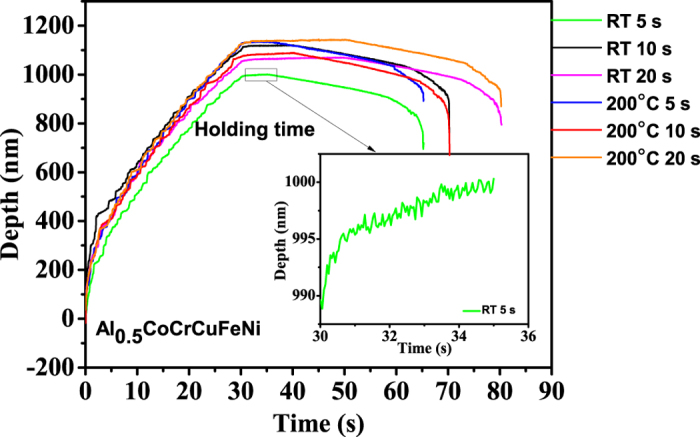
Depth-time curves for the nanoindentation of the Al_0.5_CoCrCuFeNi HEA with the holding times of 5 s, 10 s, and 20 s at room temperature (RT) and 200 °C.

Figure 1 shows the schematic of the indentation process. At the beginning of (a), the indentation load was applied to the sample at a loading rate of 10.00 mN/s, reaching the maximum load of 100 mN at the point of (c), and then maintained at 100 mN for different times of 5, 10, and 20 s. After the holding period of (c), the indentation load was reduced at an unloading rate of 10.00 mN/s. Figure 2 shows typical displacement vs. time curves for the indentations at room temperature and 200 °C with three holding times of 5, 10, and 20 s.

## Microstructures and Methods of Analysis

**Figure 3:**
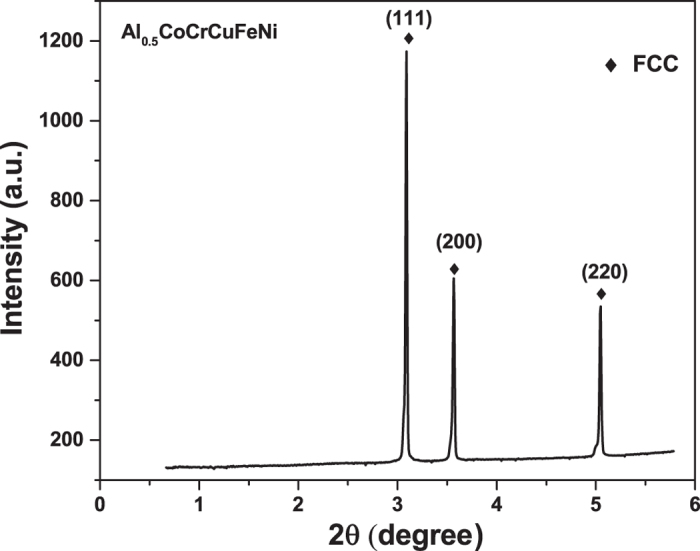
Diffraction pattern of the initial microstructure of theAl_0.5_CoCrCuFeNi HEA.

The synchrotron XRD patterns for the initial Al_0.5_CoCrCuFeNi alloy are shown in Fig. 3. The peaks in the XRD image appear as the single FCC phase with no pronounced peak splitting and texturing during the solidification process.

In the present work, the dynamical and statistical analyses are applied to investigate the evolution of serrations at the stage of a constant indentation load. First, we briefly introduce several methods for dynamical and statistical analyses to be used.

### Time-series analysis

Given a time series, {*σ*(*k*), (*k* = 1, 2, …, *N*)}, the mutual information method^42^ is used to introduce a time-delay reconstruction of a phase space. To reconstruct a phase space from a signal, a time delay and an embedding dimension are needed. For two time sequences, *A* and *B*, the probabilities of measuring values of *a*_*i*_ and *b*_*k*_ are *P*_*A*_(*a*_*i*_) and *P*_*B*_(*b*_*k*_), respectively. Note that the union probability for *a*_*i*_ and *b*_*k*_ to appear simultaneously is *P*_*AB*_(*a*_*i*_, *b*_*k*_). Defining the mutual information quantity, 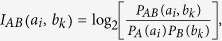 the mean mutual information quantity can be expressed as 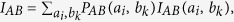 where *I*_*AB*_ represents the degree of the correlation between the sequences, *A* and *B*. There is *P*_*AB*_(*a*_*i*_, *b*_*k*_) = *P*_*A*_(*a*_*i*_)*P*_*B*_(*b*_*k*_) and *I*_*AB*_(*a*_*i*_, *b*_*k*_) = 0, if the sequence, *A*, is independent of *B*. In this study, the sequences of A and B are replaced by the signals, *σ*(*k*) and *σ*(*k* + *τ*). Then the mean mutual information quantity of 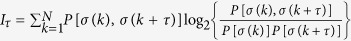 is obtained. The value of *τ*, with the minimum value of *I*_*τ*_ present at the first time, is considered as the delay.

Define*Y*_*i*_(*m*) = {*σ*(*i*), *σ*(*i* + *τ*), …, *σ*(*i* + (*m* − 1)*τ*), *i* = 1, …, [*N* − (*m* − 1)*τ*]}, where *N*is the number of serrations during the holding period, and *i* is the *i*-th evolution. The Cao method^43^ is used in the computation of the embedding dimension (usually, we called the minimum dimension of which the manifold can embed into the space as the embedding dimension).

Let 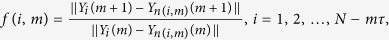 where *f*(*i*, *m*) represents the relative change of the module of the nearest event, and ||·|| is defined as Euclidian module with 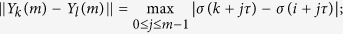 *n*(*i*, *m*) (1 ≤ *n*(*i*, *m*) ≤ *N* − *mτ*) is an integer such that *Y*_*n*(*i*,*d*)_ is the nearest event of *Y*_*i*_ according to the module ||·||. Define 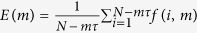, and *E*1(*m*) = *E*(*m* + 1)/*E*(*m*) as the mean value of all *f*(*i*, *m*) and the variation from *m* to *m* + 1, respectively. For a given sequence, one can determine the critical value, *m*_0_, when *E*1(*m*) remains the same. Note that it is difficult to determine *m* if *E*1(*m*) is slowly increasing or *m* is sufficiently large in computation. To confirm the embedding dimension, the other quantity is needed. Let 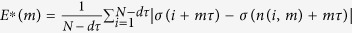, and *E*2(*m*) = E^*^(*m* + 1)/E^*^(*m*). For a deterministic data, there always exists some *m* with *E*2(*m*) ≠ 1. The value of *m* is, then, determined when *E*1(*m*) is unchanged, and *E*2(*m*) is approaching 1.

### Largest Lyapunov exponent

After the delay and embedding dimension are calculated, the Wolf’s method^44^ is used to find the largest Lyapunov exponent. For detailed analysis, see refs 45, 46, 47, 48. For a given time series, the set of 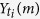  = {*σ*(*t*_*i*_ + *τ*), …, *σ*(*t*_*i*_ + (*m* − 1)*τ*), *t*_*i*_ = 1, …, [*N* − (*m* − 1)*τ*]} constitutes the reconstructed attractor with the delay, *τ*, and the embedding dimension, *m*.

Starting from the initial point, *Y*(*t*_0_), and its nearest neighbor point,*Y*_0_(*t*_0_)[the European distance between these two points, *L*(*t*_0_)], one can track the evolution of these two points until a time, *t*_1_, with 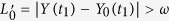, is obtained. Here *ω* is a constant and slightly larger than the minimum distance of each two points. Then the point, *Y*_1_(*t*_1_), is the nearest neighbor point to*Y*(*t*_1_). The distance between these two points is *L*_1_. Repeat the above process until the*m*-dimensional vector, *Y*(*t*_*i*_), reaches the end of the time series. Defining *M* as the total number of the repeated steps, and *t*_*M*_ as the end time, one obtains the largest Lyapunov exponent as 
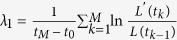
.

### Approximate entropy

Here, the concept of the approximate entropy^49^ is introduced to the system. Given the depth signal, the m-dimensional vector is defined by *Y*(*i*) = {*σ*(*i*), *σ*(*i* + 1), …, *σ*(*i* + *m* − 1), *i* = 1, …, [*N* − (*m* − 1)]},where *m* denotes the dimension. Defining the distance between *Y*_*i*_ and *Y*_*j*_ as 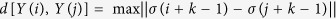, *k* = 1, 2, …, *m*, one obtains the number, *N*(*i*), of which *Y*(*i*) satisfies the condition of *d*[*Y*(*i*), *Y*(*j*)] < *r*, *j* = 1, 2, …, *N* − *m* + 1. In the calculation, *r* = 0.2*S*_*x*_ is used with *S*_*x*_ being the standard deviation of the sequence, {*σ*(*k*), (*k* = 1, 2, …, *N*)}. Let.., where 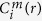 denotes the probability of *Y*_*i*_ with *d*[*Y*(*i*), *Y*(*j*)] < *r*. Denoting 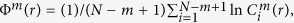 which represents the average degree of self-correlation, Φ^*m*^(*r*) − Φ^*m*+1^(*r*) represents the degree of randomness of the sequences, {*σ*(*i*), *i* = 1, 2, …, *N*}. For the reconstructed phase space, the points become rare, and the probability of correlations becomes small with increasing the dimension, *m*. Thus, 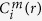 and Φ^*m*^(*r*) decrease with a larger value of *m*. The approximate entropy is calculated as *ApEn* = Φ^*m*^(*r*) − Φ^*m*+1^(*r*).

### Fractal dimension

In this section, the box-counting method in^50^ is introduced. To cover the total data of a segment with a length, *L*, one needs at least *N*(*l*) = *L*/*l* boxes. Similarly, to cover the total data of a square with a side length of *L*, one needs at least *N*(*l*) = (*L*/*l*)^2^ boxes. Changing the box size of *l*, one obtains a series of *N*(*l*). Using the induction, the measurement can be expressed as *N*(*l*) = ((*N*)/(*l*))^*D*^, where *D* is the fractal dimension of the signal. Taking natural logarithm processing of the above expression, we obtain *D* = ln *N*(*l*)/(ln *L* *− *ln *l*) If *l* is small enough so that −ln *l* ≫ ln *N*, and 
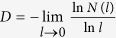
 is obtained. For the slip bands^12^,^22^,^39^, there exists a fractal structure due to the interactions among the hierarchies of slip bands at different positions and in different directions. A large fractal dimension suggests the concurrent nucleation of a large number of slip bands throughout the material^51^.

### Hurst exponent

The detrended fluctuation analysis, measuring the scaling behavior of the fluctuations, has been described in refs 52, 53, 54, 55. Divide the signal {*σ*(*i*), *i* = 1, 2, …, *N*, into *N*_*q*_ zones, where *N*_*q*_ = *N*/*q*, with each zone containing *q* elements. In the *k-th* zone, the detrended time series are 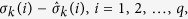 with the mean-square error, 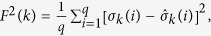 where the linear function, 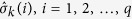, represents the local trend. The root-mean square in total *N*_*q*_ zones is calculated as 
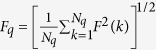
. Changing the interval length of *q* and repeating the above process, one obtains a sequence of *F*_*q*_ with *F*_*q*_ following a power-law scaling as: *F*_*q*_ ~ *q*^*H*^. Here, *H* is the Hurst exponent, which reflects the long-range memory dependence of the signal, and can predict whether the future trend is consistent with the previous or not^51^.

## Dynamic Analysis and Modeling

**Figure 4:**
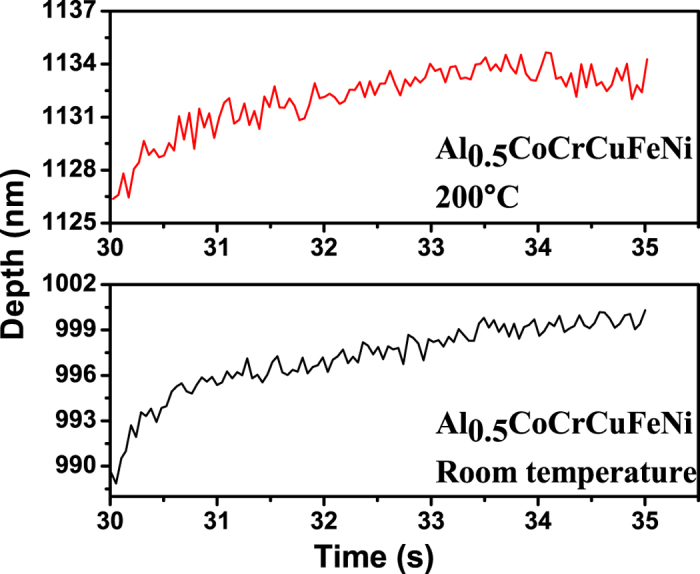
Depth-time curves at the holding phase for the nanoindentation of the Al_0.5_CoCrCuFeNi HEA with the holding time of 5 s at room temperature and 200 °C.

**Figure 5:**
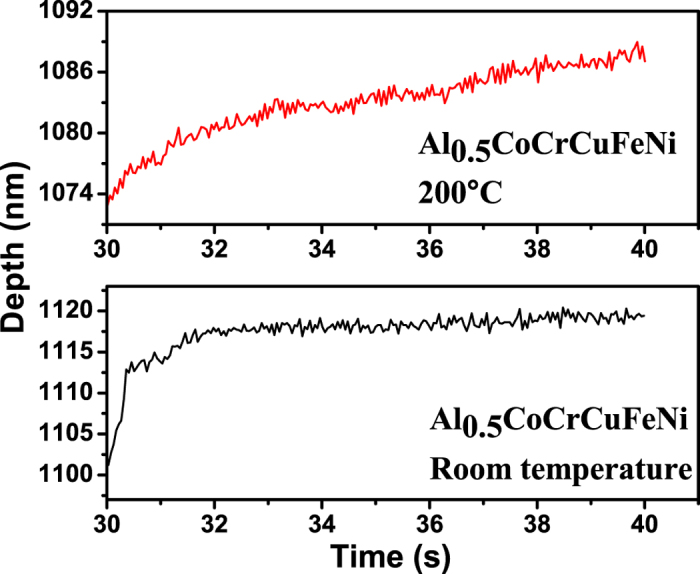
Depth-time curves at the holding phase for the nanoindentation of the Al_0.5_CoCrCuFeNi HEA with the holding time of 10 s at room temperature and 20 °C.

**Figure 6:**
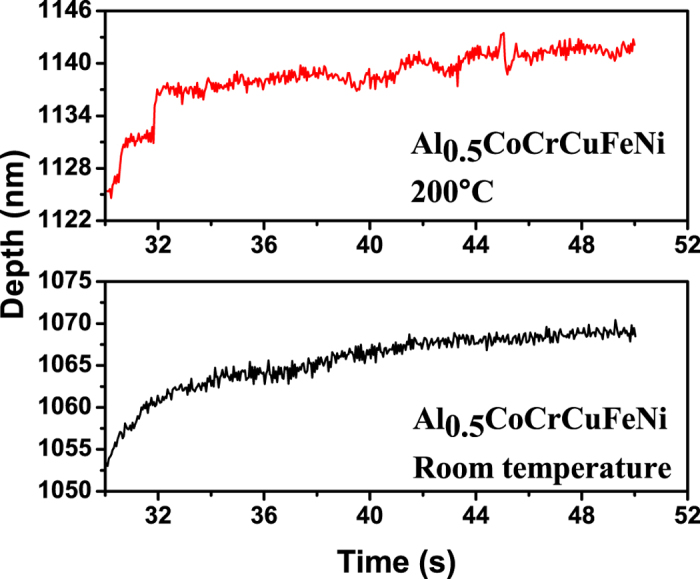
Depth-time curves at the holding phase for the nanoindentation of the Al_0.5_CoCrCuFeNi HEA with the holding time of 20 s at room temperature and 200 °C.

Consider the evolution of the serrations during the nanoindentation of the HEA, Al_0.5_CoCrCuFeNi. To examine the effect of holding time, the depth signal during the holding stage for the indentation tests, which is depicted in Figs 4, 5, 6, is used.

Using above methods of the dynamical and statistical analyses, the time delay, *τ*, embedding dimension, *m*, the largest Lyapunov exponent, *λ*_1_, the fractal dimension, *D*, Hurst exponent, *H*, and *D* + *H* of the serrated flow sequence are calculated for three holding times at room temperature and 200 °C, respectively. Tables 1 and 2 list the numerical results.

In a certain temperature and strain-rate regime, HEAs deform via sudden slips that are visible as stress drops or “serrations” in the stress-strain curves^12^. The magnitude of the stress decreases with time once a slip band is formed. The appearance of slip bands are controlled by thermally-activated rearrangements of dislocation structures^56^. The intermittent serrated flow has been recognized as a stick–slip behavior. The stick-slip behaviors are characterized by a stick phase that normally lasts much longer than the slip phase, a feature has been observed in the serration deformation of bulk metallic glasses^45^.

The stick–slip process is also a deterministic nonlinear phenomenon. The time-series analysis can offer a platform to analyze this phenomenon and shed light on the complexity of the stick-slip process. One of the characteristics for chaotic behavior is the sensitivity to initial conditions, which is quantified by the existence of a positive Lyapunov exponent, *λ*_1_. The trajectories for different initial conditions will be convergent, if the largest Lyapunov exponent is negative, while the trajectories will disperse finally for a positive Lyapunov exponent.

**Figure 7:**
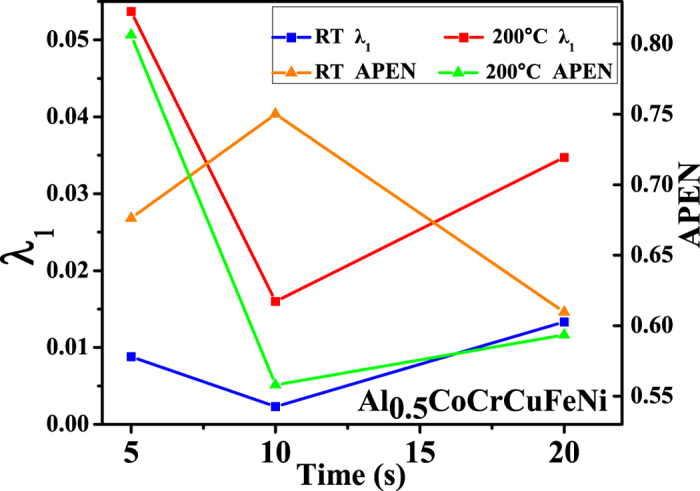
The largest Lyapunov exponent, *λ*_1_, and approximate entropy, *ApEn* for the holding times of 5 s, 10 s, and 20 s.

The largest Lyapunov exponent as a function of holding time is shown in Fig. 7. From Tables 1 and 2, the largest Lyapunov exponents are positive, regardless of temperature and holding time. This result suggests that the serration associated with slip bands possesses the complexity of chaotic behavior. The chaotic characteristics related to the formation of a single slip band likely involve the concurrent slip motion of multiple dislocations under the action of the external stress, resulting in reasonable ductility.

Note that the largest Lyapunov exponents at 200 °C are larger than those at room temperature for the same holding time, which reveals the temperature dependence of the largest Lyapunov exponent (Tables 1 and 2). At elevated temperatures, dislocations can readily move, and the dislocation motion becomes more chaotic. Figures 4, 5, 6 show that for higher temperatures, the serrations of depth-time curves become relatively larger regardless of the holding times, which are also consistent with the largest Lyapunov exponent predictions. The trend of the largest Lyapunov exponent is not a monotonous function of the holding time with an inflection at the holding time of 10 s for both temperatures. In Fig. 7, the largest Lyapunov exponent for the holding time of 10 s at room temperature is the smallest among all the values, indicating that the serrated flow during the indentation exhibits the least degree of chaos. The largest Lyapunov exponent for the holding time of 5 s at 200 °C is the greatest, suggesting that the serrated flow has the greatest degree of chaos. This result reveals that there exist interactions among dislocations, which controls the formation of slip bands. In this stage, the relaxation time approached the reloading time. The internal energy is not totally relaxed and favors the formation of new bands nearby the previous one.

The variation of the approximate entropy (ApEn) with time reflects the level of uncertainty with time. A larger value of ApEn represents more complexity and irregularity of the signal. Figure 7 also shows the variation of the approximate entropy with holding time. For the indentation at 200 °C, the trend of the approximate entropy is consistent with the largest Lyapunov exponent. At the holding time of 5 s, the largest Lyapunov exponent and approximate entropy are the largest, which suggests the increase of the interaction among slip bands and the complexity of the slip process. It is interesting to note that the tendency of the approximate entropy is in contrast to the tendency of the largest Lyapunov exponent for the indentation at room temperature. For the holding time of 10 s, the largest Lyapunov exponent is the smallest, implying that the sensitivity of the signal to initial conditions decreases, while the approximate entropy is the largest, suggesting the increase of the degree of freedom.

**Figure 8:**
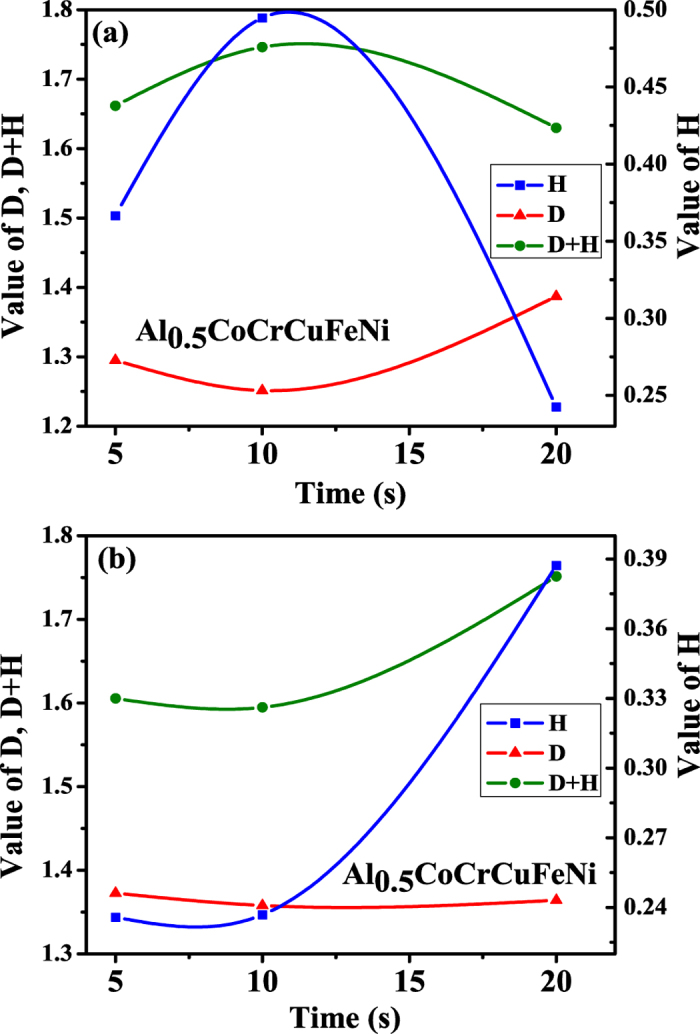
Fractal dimension, *D*, Hurst exponent, *H*, and *D* + *H* of the depth signals for the holding times of 5 s, 10 s, and 20 s; (**a**) room temperature, and (**b**) 200 °C.

The fractal dimension, *D*, which represents the fractal behavior^51^,^57^, is depicted as a function of holding time in Fig. 8. From the values of the fractal dimension shown in Fig. 8, one can conclude that the time-series exhibits a fractal behavior introduced by the long-range time correlation between the small and large fluctuations. For the indentation with the holding time of 10 s at room temperature, the fractal dimension reaches the minimum of 1.2514, which reflects a high degree of homogenization. For the holding times of 5 and 10 s, the fractal dimensions calculated for 200 °C are higher than those for room temperature, which implies that there is an enhanced fractal behavior at an elevated temperature. The largest value of *D* is 1.3871 for the indentation with the holding time of 20 s at room temperature, which represents the influence of the holding time on serrations. This result suggests that slip bands with a hierarchical structure can propagate in a scale-free manner, and there exists the effect of holding time on the serrated flow due to the stress-assisted motion of dislocations. The largest Lyapunov exponent and the fractal dimension reach the maximum with the holding time of 5 s at 200 °C while the maximum occurs with the holding time of 20 s at room temperature. This conclusion reveals that at an elevated temperature, there is an enhanced fractal behavior with slip bands interacting with each other with less holding time, while this complex phenomenon is apparent with more holding time at a lower temperature.

Figure 8 shows the variation of the Hurst exponent with holding time. A larger value of the Hurst exponent represents the self-similar stochastic behavior, while a smaller value reveals the clutter intersecting during the slip process. The Hurst exponent of an anti-persistent process is in a range of 0 to 0.5, which suggests that the evolution trend of the slip process is opposite to the past progress due to the absence of the long-memory dependence^51^. For *H* in the range of 0.5 to 1, the Hurst exponent represents a positive correlation, implying that the evolution trend of the signal is consistent with the past. The Hurst exponent of 0.5 implies that the behavior of the series is completely random, and there is no consistent relationship between past and future trends.

From Tables 1 and 2, one can note that the Hurst exponent is in the range of 0.23 to 0.49. Accordingly, the evolution trend is opposite to the past progress. The depth signal increases during a certain time and then decreases during the next time. This ascending-descending trend is consistent with the fluctuation in serration, where the serration increases with the energy aggregation and then decreases with the energy release. The Hurst exponent for the indentation at room temperature reaches the maximum of 0.4947 for the holding time of 10 s, suggesting that the depth signal is a self-similar random process with a weak negative correlation. The slip process is similar to a random walk, which introduces the homogenization of microstructures to some degree. On the other hand, the minimum of the Hurst exponents is 0.2357 for the indentation with the holding time of 5 s at 200 °C. There exists a strong negative correlation of the slip process, when the anti-persistent behavior is accompanied with the increase of the interaction of slip bands (dislocations) at an elevated temperature.

For a stochastic process, there is a linear relationship (*D* = *n* + 1 − *H*) between the fractal dimension and the Hurst exponent. Following ref. 58, a modified Cauchy class associated with the fractal dimension with the Hurst exponent is introduced, i.e., *c*(*r*) = (1 + |*r*|^*α*^)^−(*β*/*α*)−1^[1 + (1 − *β*)|*r*|^*α*^], *r* ∈ *R*, where *α* ∈ (0, 2], *β* ≥ 0.For this correlation function, the fractal dimension, *D*, and the Hurst exponent, *H*, are defined as *D* = *n* + 1 − *α*/2 and *H* = 1 − *β*/2, respectively. Here *n* is the dimension of the signal space. The correlation becomes more applicable with the modified Cauchy class, since the linear relationship is unsatisfactory. For the indentation with the holding time of 10 s at room temperature, the maximum value of *D* + *H* is 1.7461, with *α* = 1.4972, *β* = 1.0106, and *H* = 0.4947 (Table 1). The slip process is near a self-similar random process with a weak negative correlation. Note that all the values of *H* fall into (0, 0.5). One can have the restriction of *β* > 1 in the modified Cauchy class similar to the result in^51^.

For the indentation with the holding time of 20 s at 200 °C, *D* + *H* is 1.7514 (Table 2), implying a self-similar random process. For the holding time of 5 s, the Hurst exponent of 0.2357 suggests a strong negative correlation. This strong anti-persistent behavior is accompanied by the increase of the interaction among slip bands. This result is consistent with the above analysis that the largest Lyapunov exponent and the fractal behavior. Under such a condition, the plastic deformation due to multiple interactions among slip bands is characterized by a low degree of homogenization, i.e., heterogeneity. The heterogeneity in the depth signal reveals a disordered and complex slip process.

## Summary

In summary, the dynamic plastic behavior of the Al_0.5_CoCrCuFeNi HEA during the nanoindentation at both room temperature and 200 °C is chaotic. The fractal dimension and the largest Lyapunov exponents of the depth signal increase with the increase of temperature, while there is an inflection with the holding time of 10 s at the same temperature. A larger fractal dimension suggests a greater slipping rate of slip bands, which are accompanied by the spread of the hierarchical structure. It suggests that slip bands with a hierarchical structure can propagate in a scale-free manner, and there exists the effect of holding time on the serrated flow due to the stress-assisted motion of dislocations. For the indentation with holding time of 10 s at room temperature, *H* = 0.4947, and the slip evolves as a self-similar random process with a weak negative correlation.

## Additional Information

**How to cite this article**: Chen, S. *et al*. Self-Similar Random Process and Chaotic Behavior in Serrated Flow of High Entropy Alloys. *Sci. Rep.* **6**, 29798; doi: 10.1038/srep29798 (2016).

## Figures and Tables

**Table 1:** Dynamic parameters for the nanoindentation with three holding times at room temperature.

Holding Time(s)	5 s	10 s	20 s
*τ*	1	1	1
m	10	10	10
*λ*_1_	0.0088	0.0023	0.0133
*ApEn*	0.6762	0.7501	0.6096
*D*	1.295	1.2514	1.3871
*H*	0.3665	0.4947	0.2426
*D* + *H*	1.6615	1.7461	1.6297

Time delay: *τ*, embedding dimension: *m*, the largest Lyapunov exponent: *λ*_1_, approximate entropy: *ApEn*, fractal dimension: *D*, and Hurst exponent: *H*.

**Table 2:** Dynamic parameters for the nanoindentation with three holding times at 200 °C.

Holding Time(s)	5 s	10 s	20 s
*τ*	2	1	1
m	11	10	10
*λ*_1_	0.0537	0.016	0.0347
*ApEn*	0.8062	0.5581	0.5934
*D*	1.3727	1.358	1.3624
*H*	0.2357	0.2368	0.3872
*D* + *H*	1.6054	1.5948	1.7514
